# Heart in a knot: unraveling the impact of the nested tori myofiber architecture on ventricular mechanics

**DOI:** 10.1007/s10237-025-01995-y

**Published:** 2025-09-03

**Authors:** Kasra Osouli, Francesco De Gaetano, Maria Laura Costantino, Mathias Peirlinck

**Affiliations:** 1 Department of Chemistry, Materials and Chemical Engineering, Politecnico di Milano, Milan, Italy; 2 Department of BioMechanical Engineering, Faculty of Mechanical Engineering, Delft University of Technology, Delft, The Netherlands

**Keywords:** Cardiac fiber architecture, Fiber reconstruction, Rule-based methods, Nested tori, Cardiac mechanics, Computational modeling, Soft robotics

## Abstract

The intricate three-dimensional organization of cardiac myofibers and sheetlets plays a critical role in the mechanical behavior of the human heart. Despite extensive research and the development of various rule-based myofiber architecture surrogate models, the precise arrangement of these structures and their impact on cardiac function remain subjects of debate. In this study, we present a novel myofiber architecture surrogate inspired by Streeter’s nested tori conjecture, modeling the left ventricle as a series of smoothly twisting toroidal surfaces populated by continuous myofiber and sheetlet fields. Leveraging high-fidelity cardiac computational modeling approaches, we systematically evaluated the biomechanical performance of this nested tori architecture against conventional rule-based nested ellipsoidal models. Our results demonstrate that the nested tori architecture aligns more closely with experimental data on physiological myofiber and sheetlet angles. Notably, it enhances sheetlet mobility—a key mechanism for effective cardiac pumping—resulting in higher ejection fraction, greater global deformation, and a more physiological wall rotation pattern. Additionally, it produces a more homogeneous myofiber stress distribution and increased myofiber shortening during ejection. These findings suggest that the nested tori architecture provides a compelling alternative to conventional nested ellipsoidal models, offering a more physiologically consistent representation of myocardial structure and its functional implications. By enabling improved biomechanical performance in silico, this approach supports further investigation into how detailed myoarchitectural continuity shapes cardiac function. Ultimately, it may open promising avenues for advancing cardiac diagnosis, guiding the design of bioinspired implants and devices, and deepening our understanding of both healthy and diseased cardiac mechanics.

## Introduction

Cardiac computational models are increasingly recognized in clinical settings for their ability to significantly enhance the understanding of cardiac function and promote new therapies and patient-specific medical interventions (Peirlinck et al. 2021; Niederer et al. 2018). A comprehensive understanding of the ventricular myoarchitecture is imperative for accurately simulating the mechanical functions of the heart (Wilson et al. 2022; Holz et al. 2023). Cardiomyocytes assemble into layered structures known as sheetlets, each comprising clusters typically three to six cells thick. The sliding or shear deformation among sheetlets is crucial for ventricular wall thickening during systole (Wilson et al. 2022). Despite their functional importance, the precise spatial arrangement and orientation of sheetlets remain poorly characterized, largely due to methodological limitations. This uncertainty has led to persistent discrepancies in defining sheetlet directions across computational cardiac models.

Over the past few decades, the heart’s complex orthotropic microstructure has been investigated using histological data (LeGrice et al. 1995; Scollan et al. 1998; Pope et al. 2008; Hales et al. 2012) and the three-dimensional architecture of ventricular myofibers has been studied using diffusion tensor imaging acquisitions (Scollan et al. 1998; Hales et al. 2012; Nielles-Vallespin et al. 2017). Even though diffusion tensor magnetic resonance imaging offers valuable structural insights into the spatial organization of myofiber and sheetlet arrangements in the heart, the technique remains mostly applied to preclinical ex vivo research settings (Sack et al. 2018). Lengthy imaging protocols and high signal-to-noise ratios render these techniques inadequate to reconstruct precise patient-specific in vivo myofibers architectures (Piersanti et al. 2021). To overcome these issues and provide realistic in vivo myofiber architecture surrogates, atlas-based (Lombaert et al. 2012; Hoermann et al. 2019; Roney et al. 2020) and rule-based methods (Guccione et al. 1995; Rijeken et al. 1997; Franzone et al. 1998; Bayer et al. 2012; Pravdin et al. 2013; Wong and Kuhl 2014; Piersanti et al. 2022) have been proposed. Due to the intrinsic dependency and sensitivity of atlas-based methods on complex registration algorithms, rule-based methods are more common in the computational cardiac modeling field. More specifically, these methods assign either solely the myofiber’s helical and transverse angles (Guccione et al. 1995; Kerckhoffs et al. 2003; Ubbink et al. 2006; Bovendeerd et al. 2009; Pluijmert et al. 2017) or both myofiber and sheetlet orientations, treated as geometrically independent (Rossi et al. 2014; Göktepe et al. 2014; Carapella et al. 2014; Gültekin et al. 2016; Quarteroni et al. 2017; Peirlinck et al. 2019; Guan et al. 2019; Dedè et al. 2021; Holz et al. 2023; Hirschvogel et al. 2017; Pfaller et al. 2019; Eriksson et al. 2013; Zheng et al. 2023) based on transmural and apicobasal gradients and normalized distances across the computational domain. While these rule-based methods can approximate myofiber and sheetlet orientations throughout the myocardium, accurately preserving the three-dimensional continuity of these structures across the transmural depth and along the apicobasal axis remains challenging. This difficulty arises from the inherent complexity of smoothly transitioning fiber orientations and sheetlet arrangements, especially near critical regions such as the ventricular apex. These approaches naturally result in smooth nested ellipsoidal myofiber architectures, where tuning of transmural and apicobasal variation rules can be used to fit crude diffusion tensor imaging-based observations. Nevertheless, all these approaches result in singularities at the apex, where fiber orientations converge to a single point. Such a myofiber architecture is in strong contrast with various studies that explored and confirmed the continuity of myofibers between the inner and outer layers of the ventricles (Peskin 1989; Jouk et al. 2000, 2007; Jouk and Usson 2021), as originally described by Streeter’s nested tori model (Streeter 1979).

The influence of the inner-to-outer continuity of the myocardial microstructure on ventricular mechanics remains poorly understood in cardiac research. In this study, we unveil a novel method to generate surrogate myofiber architectures that seamlessly integrate nested tori myofiber continuity across the ventricular wall. Harnessing computational modeling, we reveal the impact that such a non-singular and continuous myofiber architecture has on ventricular mechanics.

## Methods

### Myofiber architecture surrogate construction

#### Nested tori (NT) architecture

**Fig. 1:**
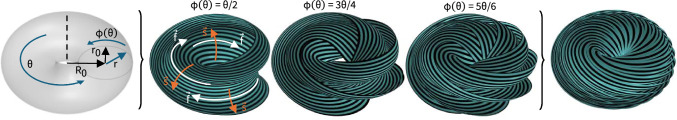
**Parametric surfaces of nested closed curves within a circular torus**. The left panel shows the toroidal coordinate system and its corresponding parameters. The middle panels show the results of modifying the $\phi (\theta )$ functions to generate diverse twisted surfaces (green) of nested torus knots (black stripes) within the toroidal domain. The direction of the vector $\varvec{f}$ is aligned with the torus knots (white arrows), while the vector $\varvec{s}$ is transversely oriented on the sheets as shown by the orange arrows. Due to the axial symmetry, the surface is repeated around the axis of revolution by rotating it in 10-degree increments. This process ensures that the entire toroidal space is represented, as illustrated in the right panel

To define the myoarchitecture in accordance with Streeter’s conjecture, we approximate the left ventricular geometry to be an ellipsoidal torus. As shown in Fig. 1, we first define a torus with minor and major radii of $r_{0}$ and $R_{0}$, respectively. Within this torus domain, we create a parametric surface of nested closed curves, so-called torus knots,

1 $$\begin{aligned} \varvec{t}\left( r, \theta \right) = \langle x(r,\theta ),y(r,\theta ),z(r,\theta ) \rangle \end{aligned}$$

where the component functions *x*, *y*, and *z*, are real-valued functions of the inner radius *r* ranging $[0,r_{0}]$, $\theta$ represents the rotation around the torus’ major axis of revolution, and the rotational function $\phi (\theta )$ specifies the rotation cadence around the circular profile of the torus.

2 $$\begin{aligned} \begin{pmatrix} x(r,\theta ) \\ y(r,\theta ) \\ z(r,\theta ) \end{pmatrix} = \begin{pmatrix} R_{0} \sin (\theta )+r \cos (\phi (\theta ))\sin (\theta ) \\ R_{0}\cos (\theta )+ r\cos (\phi (\theta ))\cos (\theta ) \\ r\sin (\phi (\theta )) \end{pmatrix} \end{aligned}$$

Depending on the choice of the rotational cadence $\phi (\theta )$, the parametric surface $\varvec{t}$ generates twisted surfaces resembling a Möbius strip, composed of a collection of curves that form various nested torus knots. Figure 1 illustrates the resulting torus knot configurations for rotational cadence functions $\phi (\theta ) = \theta /2$ with $\theta$ ranging $[0,4\pi ]$, $\phi (\theta ) = 3\theta /4$ with $\theta$ ranging $[0,8\pi ]$, and $\phi (\theta ) = 5\theta$/6 with $\theta$ ranging $[0,12\pi ]$, respectively. After evaluating various rotational cadence functions, we found that selecting $\phi (\theta ) = 5\theta /6$ yields a physiologically realistic transmural helix angle variation ranging from approximately $+60^\circ$ at the endocardium to $-60^\circ$ at the epicardium. Therefore, we set $\phi (\theta ) = 5\theta /6$ with $\theta$ ranging $[0,12\pi ]$ and leverage the $\theta$- and *r*- directional gradients to define localized fiber $\varvec{f}_{tor}$ and sheet $\varvec{s}_{tor}$ directions on these nested tori surfaces,

3 $$\begin{aligned} \begin{aligned} \varvec{f}_{tor}&= \nabla _{\theta } \, \varvec{t}\left( r, \theta \right) \\ \varvec{s}_{tor}&= \nabla _{r} \, \varvec{t}\left( r, \theta \right) \end{aligned} \end{aligned}$$

Through a rotational repetition of the nested tori surfaces in 10-degree increments around the major axis of revolution, we define localized fiber $\varvec{f}_{tor}$ and sheet $\varvec{s}_{tor}$ directions in the whole toroidal domain, as shown in the right panel of Fig. 1.

**Fig. A1:**
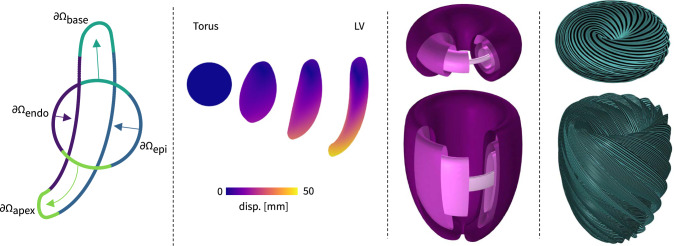
**Mapping the nested tori architecture from a circular torus to the left ventricular geometry**. We set up a Dirichlet-based mechanical deformation analysis to compute the nonlinear deformation map $\varvec{x} = \varvec{\varphi }_{tor2lv}(\varvec{X}): \varOmega _{tor} \rightarrow \varOmega _{lv}$. More specifically, we label the boundaries of the axi-symmetric slice of the left ventricle and the torus such that $\partial \varOmega _{lv/tor}=\partial \varOmega _{epi}\cup \partial \varOmega _{base}\cup \partial \varOmega _{endo}\cup \partial \varOmega _{apex}$ (first panel). As part of our approach, we intentionally introduce a small opening at the apex of the ventricle to render the domain homomorphic to a torus. This modification allows us to morph the torus into the ventricular shape without excessive geometric distortion or singularities near the apex. We derive a continuous displacement field by computing the difference between the coordinates of points on the torus boundaries and those on the left ventricle boundaries. To find the deformation map, we solve a mechanical equilibrium boundary value problem $\nabla . \varvec{(FS)} = 0$ in $\varOmega _{tor}$, with Dirichlet-based boundary conditions $\varvec{u|_{\varGamma }} = \varvec{u_{0}}$ on $\partial \varOmega _{tor}$. Here, this is represented as $\varvec{u_{0}}=\partial \varOmega _{lv}-\partial \varOmega _{tor}$. We impose a simple compressible Neo-Hookean strain energy density function $\psi = \frac{1}{2}\left( \mu (I_{1}-3)+\lambda (J-1)^2 \right)$ to the domain with Lamé parameters $\mu$ and $\lambda$ have values of 1 Pa and 300 Pa, respectively. The nonlinear deformation map $\varvec{\varphi }_{\textrm{tor2lv}}$ can be written as a decomposition of the resulting deformation gradient tensor. Specifically, we adopt the polar decomposition $\varvec{F} \;=\; \varvec{R}\,\varvec{U}$, where $\varvec{R}$ is an orthogonal tensor representing rigid-body rotation, and $\varvec{U}$ is the symmetric right stretch tensor describing local deformation. Since only the fiber orientation is of interest, we retain the rotation tensor $\varvec{R}$ as our deformation map $\varvec{\varphi }_{\textrm{tor2lv}}$ and disregard the stretching effects captured by $\varvec{U}$. Boundary labels of the axi-symmetric slice of the left ventricle and the torus are shown in the first panel on the left. The resulting nonlinear deformation map $\varvec{\varphi }_{tor2lv}$ is shown in the second panel. The third panel shows the nested toroidal surfaces on which we define the closed curves, in different shades of purple before and after being morphed into the shape of the left ventricle. The fourth panel depicts the nested tori arrangement of fibers and sheets following the transformation of the torus into a left ventricular shape. The green surfaces represent the sheets, with the fibers indicated by black stripes

To morph the resulting toroidal fiber and sheet vector fields into ventricular fiber and sheet vector fields, we construct a nonlinear deformation map $\varvec{\varphi }^*$ which maps material points $\varvec{X}_{tor}$ from an axi-symmetric torus slice to material points $\varvec{X}_{lv}$ into an axi-symmetric slice of our ventricular geometry described in Sect. 2.2.4, i.e. $\varvec{X}_{lv} = \varvec{\varphi }^*(\varvec{X}_{tor}): \varOmega _{tor} \rightarrow \varOmega _{lv}$. Here, we adopt a pseudo-mechanical static equilibrium problem to compute an appropriate $\varvec{\varphi }^*$ map as detailed in Fig. A1. Alternatively, we can define and solve Laplace problems on both $\varOmega _{tor}$ and $\varOmega _{lv}$ and use the corresponding map to compute an appropriate $\varvec{\varphi }^*$ map. To conclude our nested myofiber architecture computation, we map our toroidal fiber and sheet directions onto their respective ventricular fiber and sheet directions,

4 $$\begin{aligned} \begin{aligned} \varvec{f}_{lv}&= \varvec{\varphi }^* \left( \varvec{f}_{tor} \right) \\ \varvec{s}_{lv}&= \varvec{\varphi }^* \left( \varvec{s}_{tor} \right) \end{aligned} \end{aligned}$$

as shown in the most right panel of Fig. A1. Due to geometric constraints, we excluded a small region at the ventricular apex from direct toroidal morphing. To maintain continuity in this region, we employed spatial interpolation techniques, thereby ensuring a smooth and continuous vector field throughout the entire ventricular geometry. While we selected specific parameters for defining the torus knots and morphing process based on initial physiological and geometric considerations, a comprehensive parameter exploration in future studies could provide deeper insights and potentially refine the nested tori architecture of myofibers and sheetlets.

#### Nested ellipsoidal (NE) architecture

**Fig. 2:**
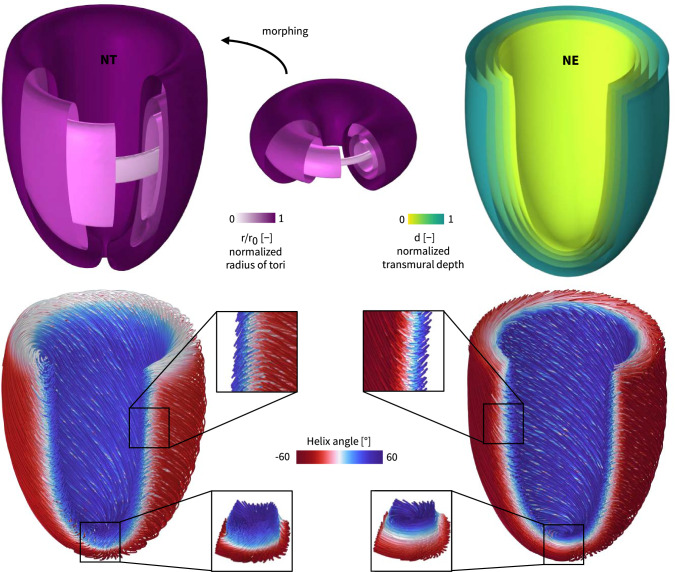
**Nested toroidal vs. ellipsoidal myofiber architectures**. The upper panel shows the resulting nested tori *NT* and nested ellipsoidal *NE* surfaces within the ventricular wall. We highlight the normalized radius of the nested tori ($r/r_0$) in the top left panel, with zero being the radius of the degenerate torus and one the outermost torus. In the top right panel, we showcase the nested ellipsoidal normalized transmural depth evolution. In the lower panel, myofibers in the left ventricle with nested toroidal *NT* (left) and nested ellipsoidal *NE* (right) myoarchitectures are illustrated as streamlines. Here, the colorbar represents the transmurally varying myofiber helix angle. Whereas the apex of the nested ellipsoidal *NE* myofiber surrogate model results in a singularity, the nested toroidal *NT* surrogate model reveals a vortex connecting the myofibers of the epicardial layers to the endocardial myofibers. In line with Streeter’s conjecture (Streeter et al. 1969), this myofiber continuity between inner endocardial and outer epicardial tissue layers occurs at different apicobasal levels

In contrast with our nested tori approach above, we adopt the Laplace–Dirichlet rule-based method (Bayer et al. 2012; Wong and Kuhl 2014) to generate nested ellipsoidal *NE* myoarchitectures within our ventricular domain. This method assumes that the fiber direction $\varvec{f}$ varies as a function of the normalized transmural depth *d* and remains parallel to the endocardial and epicardial surfaces, resulting in the nested ellipsoidal surfaces shown in Fig. 2. Following this framework, we prescribe a linear variation in the fiber helix angle from $+60^{\circ }$ at the endocardium to $-60^{\circ }$ at the epicardium (LeGrice et al. 1995; Guccione et al. 1995; Streeter et al. 1969). With regard to the sheet and normal directions in computational ventricle models, two principal modeling strategies can be distinguished (Aróstica et al. 2025). The first, building on the seminal work of LeGrice et al. (1995), assumes that the sheet direction $\varvec{s}$ is aligned with the transmural direction, while the normal direction $\varvec{n}$ is orthogonal to both fiber and sheet directions (Rossi et al. 2014; Göktepe et al. 2014; Quarteroni et al. 2017; Peirlinck et al. 2019; Guan et al. 2019; Dedè et al. 2021; Holz et al. 2023). Because the sheetlets in this case are oriented perpendicular to the endo- and epicardial surfaces, we refer to this configuration as the $NE_{\perp }$ approach. Alternatively, other studies (Hirschvogel et al. 2017; Pfaller et al. 2019) define the normal direction $\varvec{n}$ along the transmural direction, with the sheet direction $\varvec{s}$ orthogonal to both fiber and normal directions. We denote this configuration as the $NE_{||}$ approach.

### Cardiac mechanics

#### Kinematics

Let $\varOmega _{0} \subset \mathbb {R}^3$ denote the reference configuration of the deformable myocardial tissue body that occupies the current configuration $\varOmega \subset \mathbb {R}^3$ at time $t \in \mathbb {R}^+$. Any material point $\varvec{X} \in \varOmega _{0}$ in the reference configuration maps to its spatial position $\varvec{x} \in \varOmega$ in the deformed state through the nonlinear deformation map $\varvec{x} = \varvec{\varphi }(\varvec{X}): \varOmega _{0} \rightarrow \varOmega$. The gradient of the deformation map $\varvec{\varphi }$ with respect to the undeformed coordinates $\varvec{X}$ defines the deformation gradient $\varvec{F}$ with its determinant *J*,

5 $$\begin{aligned} \varvec{F} = \nabla _{\varvec{X}} \, \varvec{\varphi } \left( \varvec{X} \right) \quad \text{ with } \quad J = \det (\varvec{F}) > 0, \end{aligned}$$

which we multiplicatively decompose into a volumetric part $\varvec{F}_{\text {vol}}$ and an isochoric part $\bar{\varvec{F}}$,

6 $$\begin{aligned} \varvec{F}_{\text {vol}} = J^{1/3}\varvec{I} \quad \text{ and } \quad \bar{\varvec{F}} = J^{-1/3}\varvec{F}. \end{aligned}$$

As deformation measures, we introduce the right and left Cauchy–Green deformation tensors, $\varvec{C}$ and $\varvec{b}$, and their isochoric counterparts, $\bar{\varvec{C}}$ and $\bar{\varvec{b}}$,

7 $$\begin{aligned} \begin{array}{l} \varvec{C} = \varvec{F}^{\mathrm{{t}} } \cdot \varvec{F} \\ \varvec{b} = \varvec{F} \cdot \varvec{F}^{\mathrm{{t}} } \end{array} \qquad \text{ and } \qquad \begin{array}{l} \bar{\varvec{C}} = \bar{\varvec{F}}^{\mathrm{{t}} } \cdot \bar{\varvec{F}}\\ \bar{\varvec{b}} =\bar{\varvec{F}} \cdot \bar{\varvec{F}}^{\mathrm{{t}} }. \end{array} \end{aligned}$$

We further characterize the deformation of the tissue through the isotropic deviatoric first invariant

8 $$\begin{aligned} \bar{I}_1 = [\varvec{\bar{F}}^{\mathrm{{t}} } \cdot \varvec{\bar{F}} ] : \varvec{I} \end{aligned}$$

and the anisotropic deviatoric invariants

9 $$\begin{aligned} \begin{aligned} \bar{I}_{\text {4ff}}&= \bar{\varvec{C}} : [\varvec{f}_0 \otimes \varvec{f}_0] \\ \bar{I}_{\text {4ss}}&= \bar{\varvec{C}} : [\varvec{s}_0 \otimes \varvec{s}_0] \\ \bar{I}_{\text {4fs}}&= \bar{\varvec{C}} :[\varvec{f}_0 \otimes \varvec{s}_0] \end{aligned} \end{aligned}$$

where $\varvec{f}_0$ and $\varvec{s}_0$ represent the undeformed myocardial fiber and sheet unit directions (Peirlinck et al. 2019). Here, invariants $\bar{I}_{\text {4ff}}$ and $\bar{I}_{\text {4ss}}$ take the interpretation of the squared stretches of the deformed fiber and sheet vectors, and $\bar{I}_{\text {4fs}}$ indicates the shear in the fiber sheet plane (Holzapfel and Ogden 2009; Peirlinck et al. 2019, 2024).

#### Governing equations

**Fig. 3:**
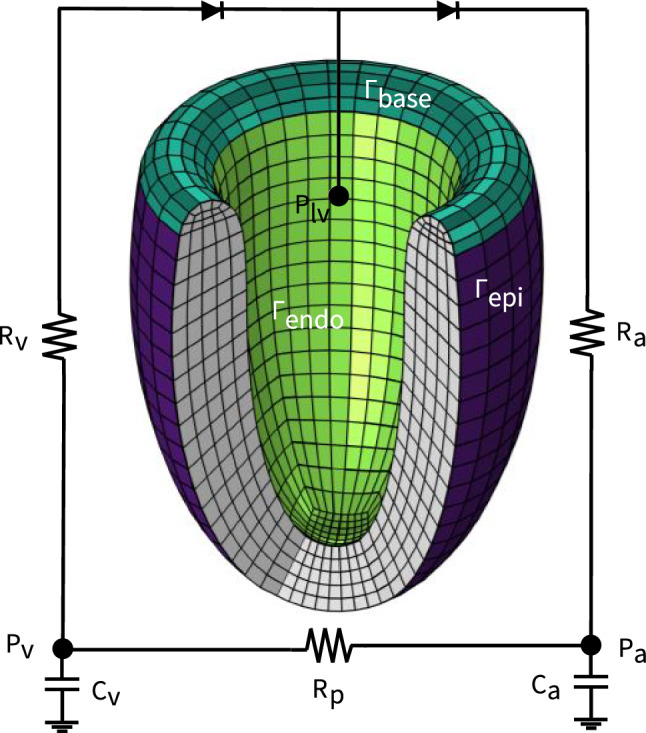
**Idealized left ventricle geometry and boundary surfaces.** The base (dark green), endocardium (light green), and epicardium (purple) are shown. Hemodynamic pressure is applied on the endocardial surface and coupled to a closed loop Windkessel model

We seek to solve the mechanical equilibrium problem on the considered domain. Assuming negligible inertial effects, the strong form equilibrium equation of our problem is:

10 $$\begin{aligned} \nabla \cdot (\varvec{F} \varvec{S}) = \varvec{0} \end{aligned}$$

where $\varvec{S}$ is the second Piola–Kirchhoff stress tensor. We define the following time-varying boundary conditions

11 $$\begin{aligned} \begin{aligned} \varvec{F} \varvec{S} \varvec{n}_0 = p_{\text {lv}} J \varvec{F}^{-t} \varvec{n}_0 \qquad&\text {on }\quad \varGamma _{endo}\\ \varvec{F} \varvec{S} \varvec{n}_0 \cdot \varvec{n}_0 + \alpha ^n_{\text {epi}} u_n + \beta ^n_{\text {epi}} \dot{u}_n = 0 \qquad&\text {on }\quad \varGamma _{epi}\\ \varvec{F} \varvec{S} \varvec{n}_0 \cdot \varvec{t}_0 + \alpha ^t_{\text {epi}} u_t + \beta ^t_{\text {epi}} \dot{u}_t = 0 \qquad&\text {on }\quad \varGamma _{epi}\\ \varvec{F} \varvec{S} \varvec{n}_0 + \alpha _{\text {base}} \varvec{u} + \beta _{\text {base}} \dot{\varvec{u}} = \varvec{0} \qquad&\text {on }\quad \varGamma _{base}\\ \end{aligned} \end{aligned}$$

where $\varvec{u}: \varOmega _0 \rightarrow \mathbb {R}^3$ represents the displacement field to be found and $\varGamma _{endo}$, $\varGamma _{epi}$, and $\varGamma _{base}$ denote the three boundary subdomains of the whole domain, i.e., $\partial \varOmega _{0}=\varGamma _{epi}\cup \varGamma _{base}\cup \varGamma _{endo}$ as shown in Fig. 3. Here, the dynamically evolving intraventricular blood pressure $p_{\text {lv}}$ acts directly on the endocardial wall and $\varvec{n}_0$ and $\varvec{t}_0$ describe the normal and tangential vectors of the unit wall vector in the reference domain, respectively. More details on the Robin-type epicardial boundary conditions we developed are described in Sect. 2.2.6.

#### Constitutive modeling

We characterize the mechanical behavior of myocardial tissue following the active stress approach (Peirlinck et al. 2019):

12 $$\begin{aligned} \varvec{S} = \varvec{S}_{pas} + \varvec{S}_{act} \end{aligned}$$

where $\varvec{S}_{pas}$ and $\varvec{S}_{act}$ denote the passive and active second Piola–Kirchhoff stress tensor, respectively. We characterize the passive myocardial tissue behavior

13 $$\begin{aligned} \varvec{S}_{pas} = \frac{\partial \psi }{\partial \varvec{{E}}} + \frac{\partial \psi _{visco}}{\partial {\dot{\varvec{{E}}}}} \end{aligned}$$

where $\varvec{{E}} = \frac{1}{2} \left( \varvec{C} - \varvec{I} \right)$ denotes the Green–Lagrange deformation tensor (Aróstica et al. 2025). We adopt the nearly incompressible Holzapfel–Ogden material model for myocardial tissue (Holzapfel and Ogden 2009; Martonová et al. 2024). Throughout this paper, we use the term *myofiber* to refer to the locally dominant orientation of cardiomyocytes, consistent with the conventions established by Holzapfel and Ogden (2009) and Sommer et al. (2015).

14 $$\begin{aligned} \begin{aligned} \psi&= \frac{a}{2b} \exp \left\{ b\left( \bar{I}_1-3\right) \right\} +\sum _{i \in \{f,s\}} \frac{a_i}{2b_i} \left( \exp \left\{ b_i \langle \bar{I}_{4ii}-1 \rangle ^2\right\} -1\right) \\&\quad +\,\frac{a_{fs}}{2 b_{fs}}\left( \exp \left\{ b_{fs} \bar{I}_{4fs}^2\right\} -1\right) +\frac{\kappa }{4}\left( J^2-1-2 \ln (J)\right) \end{aligned} \end{aligned}$$

where the Macauley bracket $\langle \circ \rangle$ denotes a rectified linear unit activation function (Peirlinck et al. 2024). We further impose a viscoelastic free energy function contribution (Pfaller et al. 2019) in the form

15 $$\begin{aligned} \begin{aligned} \psi _{visco} = \frac{\eta }{2} tr \left( \dot{\varvec{{E}}}^2 \right) \end{aligned} \end{aligned}$$

and the tissue’s active contractile behavior through the active stress tensor (Bestel et al. 2001)

16 $$\begin{aligned} \varvec{S}_{act} = \tau \left( t \right) \varvec{f_0} \otimes \varvec{f_0} \end{aligned}$$

where the time-varying elastance function $\tau \left( t \right)$ is characterized by the evolution equation

17 $$\begin{aligned} \dot{\tau }(t)=-|a(t)| \tau (t)+\sigma _0 \langle a(t) \rangle . \end{aligned}$$

Here, $\sigma _0$ denotes the maximum contractility and the activation function *a*(*t*) follows from

18 $$\begin{aligned} \begin{aligned} a(t)&:= \alpha _{\max } \cdot f(t)+\alpha _{\text {min}} \cdot (1-f(t)) \\ f(t)&:= S^{+}\left( t-t_{\text {sys}}\right) \cdot S^{-}\left( t-t_{\text {dias}}\right) \\ S^{\pm }(\varDelta t)&:= \frac{1}{2}\left( 1 \pm \tanh \left( \frac{\varDelta t}{\gamma }\right) \right) . \end{aligned} \end{aligned}$$

We disclose the used passive, viscous, and active constitutive parameters in Table A1. Given the differing myocardial tissue stiffness in vitro versus in vivo (Peirlinck et al. 2019), we adopt passive myocardial tissue parameters following Peirlinck et al.’s two-stage ex vivo to in vivo calibration approach (Peirlinck et al. 2019) based on human myocardial biaxial tensile and triaxial shear test data (Sommer et al. 2015) and Klotz’s in vivo end-diastolic pressure–volume relationship (Klotz et al. 2006). We employed the described viscous pseudo potential primarily to promote numerical stability (Pfaller et al. 2019; Chapelle et al. 2012), rather than to fully capture the viscoelastic response of myocardial tissue. To limit artificial dissipation, the viscosity coefficient was deliberately chosen to be small. While a more rigorous and thermodynamically consistent formulation may provide a more accurate representation of physiological cardiac tissue viscoelasticity (Nordsletten et al. 2021), such modeling is beyond the scope of the present study.

#### Geometry

We approximate the left ventricular geometry using an axisymmetric ellipsoid (Aróstica et al. 2025; Land et al. 2015) with a curved base. More specifically, we parameterize the domain in $\mathbb {R}^3$ for a truncated ellipsoid to satisfy the following equation for the endocardial and epicardial surfaces, respectively.

19 $$\begin{aligned} (x, y, z)= \left( r \sin (\eta ) \sin (\phi ), r\sin (\eta )\cos (\phi ), R \cos (\eta ) \right) \end{aligned}$$

where *r* and *R* serve as the minor and major radii of the endocardial (light green—Fig. 3) and epicardial (purple—Fig. 3) ellipsoids, respectively. Here, $\eta$ and $\phi \in \left[ -\pi ,\pi \right]$ denote the angular latitude and longitude, respectively. The curved base is defined using three Bezier splines, i.e.

20 $$\begin{aligned} \begin{aligned} B_{1}(\zeta )&= P_{0}(1-\zeta )^3 + 3P_{1}(1 - \zeta )^2 t+3P_{2}(1 - \zeta )\zeta ^2 + P_{3}\zeta ^3 \\ B_{2}(\zeta )&= P_{3}(1-\zeta )^3 + 3P_{4}(1 - \zeta )^2 t+3P_{5}(1 - \zeta )\zeta ^2 + P_{6}\zeta ^3 \\ B_{3}(\zeta )&= P_{6}(1-\zeta )^2 + 2P_{7}(1 - \zeta ) t + P_{8}\zeta ^2 \end{aligned} \end{aligned}$$

with $\zeta \in [0,1]$. The used geometrical parameters are summarized in Table A1.

#### Hemodynamic boundary conditions

To realistically simulate ventricular loading conditions throughout the cardiac cycle, we incorporate dynamically changing intraventricular pressures. Specifically, we couple the intraventricular pressure to a closed loop lumped parameter hemodynamic model (Kroon et al. 2009), as illustrated in Fig. 3. This reduced-order model connects the left ventricle to arterial and venous compartments via idealized diode elements, emulating valve function. These diodes permit blood outflow when ventricular pressure exceeds arterial pressure $P_{a}$ and inflow when it falls below venous pressure $P_{v}$. Arterial and venous compartments are characterized by their respective resistances $R_{a}, R_{v}$ and compliances $C_{a}, C_{v}$, and are interconnected by peripheral resistance $R_{p}$.

At the start of our simulation, we ramp the ventricular pressure gradually up to an initial target $p_{lv,i}$. Thereafter, ventricular pressure evolves dynamically, entirely determined by the Windkessel model and valve-like diode behavior. The initial pressures are set to physiological values of 70 mmHg in the arterial compartment and 8 mmHg in the venous compartment. Simulations are executed over five consecutive cardiac cycles, ensuring convergence of the pressure and boundary force responses (Fig. A3). Table A1 summarizes our hemodynamic lumped parameter model parameters.

#### Pericardial boundary conditions

The human heart is enclosed in the pericardial cavity, where the pericardium provides both spatial support and frictionless sliding of the myocardium. To account for its mechanical influence, we incorporate the pericardium in our models through Robin-type boundary conditions.

Conventional approaches typically rely on the undeformed normal and tangential vectors of the epicardial surface to prescribe these conditions. However, such reference configuration based Robin conditions (Pfaller et al. 2019; Strocchi et al. 2020) can lead to nonphysiological pericardial forces, especially under large rotations of the epicardial wall. Specifically, these methods project the spatial displacement $\varvec{u}$ onto the normal vector $\varvec{N}$ of the reference configuration to compute the gap between the epicardium and pericardium, an assumption valid only under small rotations (Pfaller et al. 2019).

To circumvent such a limitation and avoid nonphysiological epicardial restraints in the presence of large rotations, we develop an efficient geometric approach that incorporates Robin-type boundary conditions based on the current configuration.

More specifically, we leverage prolate spheroidal coordinate transformations and assume that both the deformed epicardial and reference pericardial surface can be locally described as prolate spheroids. We compute the ellipsoidal radial distance between any current material point on the epicardial surface and the reference pericardial surface in a few steps. First, we compute the material point’s radial distance $r_{xy}$ in the *xy*-plane:

21 $$\begin{aligned} r_{xy} = \sqrt{x^2 + y^2}. \end{aligned}$$

Combined with the material point’s *z*-coordinate and the assumption that both surfaces are described by ellipsoids with the same focal length

22 $$\begin{aligned} a = \sqrt{R_\text {peri}^2-r_\text {peri}^2}, \end{aligned}$$

we compute the auxiliary quantities

23 $$\begin{aligned} \begin{aligned} R&= \sqrt{(a + z)^2 + r_{xy}^2}, \\ S&= \sqrt{(a - z)^2 + r_{xy}^2}. \end{aligned} \end{aligned}$$

Lastly, we obtain the ellipsoidal radial distance $\xi$, angular coordinate $\eta$ and azimuthal angle $\phi$:

24 $$\begin{aligned} \begin{pmatrix} \xi \\ \eta \\ \phi \end{pmatrix} = \begin{pmatrix} \cosh ^{-1}\left( \dfrac{R + S}{2a} \right) \\ \arccos \left( \dfrac{R - S}{2a} \right) \\ \arctan 2(y, x) \end{pmatrix} \end{aligned}$$

We compute the reference $\varvec{X}_\text {epi} = (\xi _0, \eta _0, \phi _0)$ and current $\varvec{x}_\text {epi} = (\xi , \eta , \phi )$ epicardial surface positions with respect to the original prolate pericardial reference surface. We leverage these two coordinate positions to calculate $\varvec{\tilde{x}}_{peri} = (\xi _0, \eta , \phi )$ and the updated ellipsoidal radial vector component of the mapping between the reference pericardial surface and the current epicardial surface:

25 $$\begin{aligned} \varvec{\mu }_{\xi } = \left( \xi -\xi _0 , 0, 0 \right) \end{aligned}$$

**Fig. 4:**
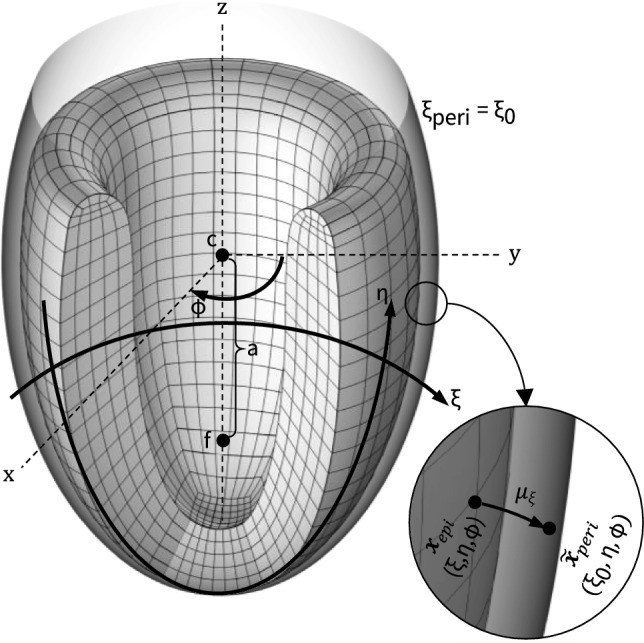
**Pericardial boundary condition based on confocal prolate spheroidal coordinate system projection**. Global Cartesian coordinates (x,y,z) and the prolate spheroidal coordinates ($\xi$, $\eta$, $\phi$) with the focal length of *a* centered at *c*. The pericardial surface is shown as a prolate spheroid (ellipsoid) with constant $\xi = \xi _0$. At each time step, any current material point $\varvec{x}_{epi}(\xi , \eta , \phi )$ on the epicardial surface is mapped onto the pericardial surface , $\varvec{\tilde{x}}_{peri}(\xi _0, \eta , \phi )$ to compute $u_n$ from $\varvec{\mu _{\xi }} = (\xi -\xi _0, 0, 0)$

We measure the Robin-type spring boundary condition length $u_n$ in Eq. 11 as the Euclidian distance of $\varvec{\mu }_{\xi }$ using the scaling factors associated with the prolate spheroidal coordinate system,

26 $$\begin{aligned} u_n = a \sqrt{\sinh ^2\left( \xi \right) +\sin ^2\left( \eta \right) }\left( \xi -\xi _0\right) \end{aligned}$$

where *a* denotes the focal length discussed above (Fig. 4). Our proposed pericardial boundary condition method performs optimally in idealized geometries, where the pericardial surface can be analytically represented as an ellipsoid. Extending this method to realistic, patient-specific cardiac geometries will require additional modeling considerations to accurately capture the complex shapes of real pericardial surfaces.

#### Solver information

We spatially discretize our computational domain using 3616 quadratic serendipity hexahedral elements with a reduced integration scheme and iteratively solve the weak form of the governing equations utilizing a backward implicit time integration approach in COMSOL v6.1.

### Performance metrics

Incorporating identical contractility and hemodynamic loading conditions across all three cases, we quantitatively compare the impact that the three differing myofiber architectures *NT*, $NE_{\perp }$, and $NE_{||}$ have on the deformation and hydraulic performance of the ventricle. More specifically, we compare the global mechanical behavior by comparing the stroke volume $SV \,$[ml] and ejection fraction $EF \, [\%]$

$$\begin{aligned} \begin{aligned} SV&:= EDV-ESV\\ EF&:= {SV}/{EDV}\\ \end{aligned} \end{aligned}$$

where *EDV* and *ESV* represent the end-diastolic and end-systolic intraventricular volume, respectively. We compare the wall thickening $WT \, [\%]$ and longitudinal shortening $LS \, [\%]$

$$\begin{aligned} \begin{aligned} WT&:= \frac{t_w-t_{w,ED}}{t_{w,ED}},\\ LS&:= \frac{a_s-a_{s,ED}}{a_{s,ED}}, \end{aligned} \end{aligned}$$

where $t_w$ and $t_{w,ED}$ represent the current and end-diastolic average thickness of the wall, respectively, and where $a_s$ and $a_{s,ED}$ denote the current and end-diastolic length between the apex and the base, respectively. We further compute myofiber architecture induced differences in myocardial stroke energy density $w \, [kJ/m^3]$

$$\begin{aligned} w = \int \varvec{S}:\ d\varvec{E} \end{aligned}$$

Here, $\varvec{S}$ denotes the second Piola–Kirchhoff stress tensor (Eq. 12) and $\varvec{E}$ denotes the Green–Lagrange deformation tensor as discussed before. We compute the volume-averaged myocardial stroke energy density across the entire ventricular domain.

Beyond global characteristics, we also study the effect that varying myofiber architectures have on local kinematic and mechanical characteristics, including the regional azimuthal rotation of the ventricular wall $\varDelta \phi \, [^\circ ]$,

$$\begin{aligned} \varDelta \phi = \arctan 2 \left( y , x \right) . \end{aligned}$$

We measure the endo- and epicardial rotation of the base and apex at the intersection points between the *xz* plane and the endo- and epicardial surfaces where *x* and *y* are the coordinates of the points at each time step. Additionally, we compare local volume-averaged myofiber stresses $\sigma _{ff} \, [kPa]$ and Green–Lagrangian myofiber strains $E_{ff} \, [-]$ in three transmural subregions: $\varOmega _{lv,endo}$ ($0\le d \le 0.1$), $\varOmega _{lv,mid}$ ($0.45\le d \le 0.55$) and $\varOmega _{lv,epi}$ ($0.9\le d \le 1$).

## Results

**Fig. 5:**
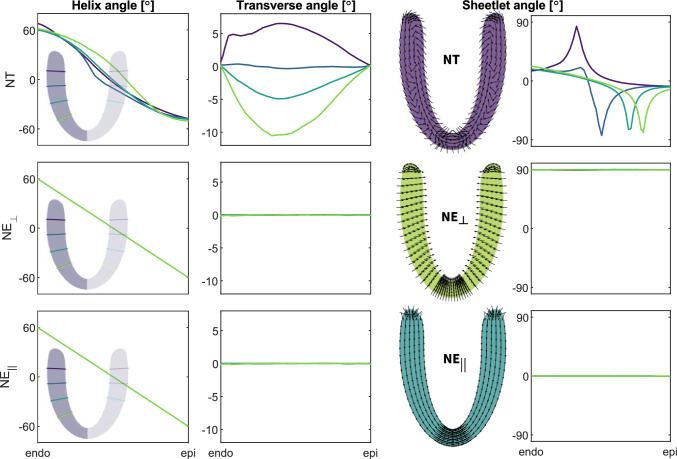
**Transmural variation in helical myofiber angles, transverse myofiber angles, and sheetlet angles across the** *NT*, $NE_{\perp }$ **and** $NE_{||}$ **myofiber architecture surrogates**. The transmural variation in helical (first column) and transverse (second column) myofiber angles is illustrated at four distinct apicobasal levels for the *NT*, $NE_{\perp }$, and $NE_{||}$ myofiber architectures. The four apicobasal levels are distinguished by four different line colors shown on the long-axis slice of the ventricle as subplot (first column). The sheetlet angle variation is shown in a axi-symmetrical longitudinal cross section (third column) and along the transmural depth at four distinct apicobasal levels (fourth column) for the *NT*, $NE_{\perp }$ and $NE_{||}$ myofiber architectures, respectively. Here, black arrows showcase the sheetlet vector variations on longitudinal sections of the left ventricle for each of the three different myoarchitectures

### Nested toroidal versus nested ellipsoidal tissue macrostructure

**Fig. A2:**
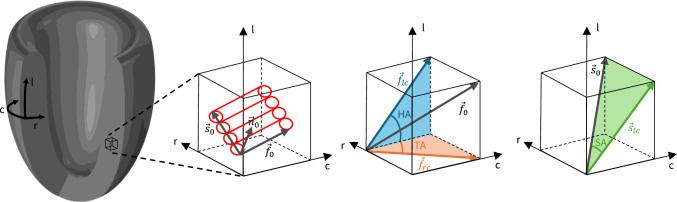
**Definition of myofiber and sheetlet angles**. On the left, the reference configuration myofiber ($\varvec{f}_0$), sheetlet ($\varvec{s}_0$), and normal ($\varvec{n}_0$) vectors are shown in a representative volume element within the ventricular wall with local coordinates: radial (r), circumferential (c), and longitudinal (l). The angle between the myofiber’s projection on the l-c plane and the circumferential direction defines the helix angle (*HA*), and the angle between the fiber projection on the r-c plane and the circumferential direction defines the transverse angle (*TA*) (middle). The sheetlet angle (*SA*) is the angle between the sheetlet vector and its projection on the l-c plane (right) (Lombaert et al. 2012; Agger and Stephenson 2020; Wilson et al. 2022)

**Fig. A3:**
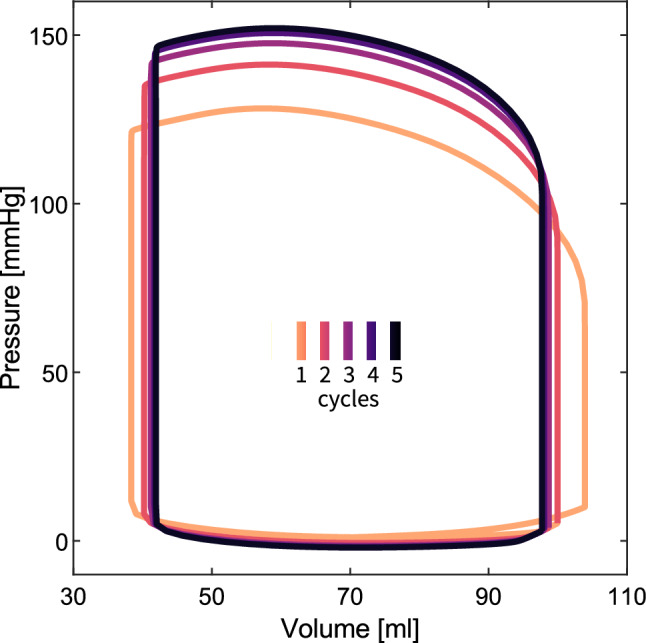
**Pressure–volume loops of the left ventricle with nested tori myofiber architecture for five cardiac cycles**. Pressure–volume loops from multiple cycles are shown in different colors, demonstrating that both pressure and volume curves reach a steady state after five cycles

Figure 5 showcases the transmurally varying helical myofiber, transverse myofiber, and sheetlet angles for the *NT*, the $NE_{\perp }$, and the $NE_{||}$ models, respectively (see Fig. Fig. A2 for detailed angle definitions). While the $NE_{\perp }$ and $NE_{||}$ helical myofiber angles follow the intrinsically assigned linear ramp from $+60^\circ$ in the endocardium to $-60^\circ$ at the epicardium, the *NT* myofiber architecture reveals a transmural S-shaped evolution of the helical myofiber angles at various apicobasal locations. Following conventional nested ellipsoidal rule-based assumptions for assigning myofiber directions, the transverse angles in the $NE_{\perp }$ and $NE_{||}$ myofiber architectures remain constant at $0^\circ$ throughout the ventricular wall. In the *NT* myofiber architecture surrogate model on the other hand, the transverse angles amount to $0^\circ$ at the endocardial and epicardial wall, but show significant variations throughout the transmural wall. For all considered apicobasal locations, we observe a gradual increase in the absolute value of the transverse angle from the endocardial surface to a maximum value at the middle of the wall, after which the absolute transverse angle drops back to $0^\circ$ in the subepicardium. Dependent on the apicobasal location, this maximum transverse myofiber angle in the midwall varies from $-10^\circ$ at the apex to $+6^\circ$ at the base. The sheetlet angles remain constant throughout the ventricular wall following the assumption for the $NE_{\perp }$ and $NE_{||}$ myofiber architectures at $90^\circ$ and $0^\circ$, respectively. However, the transmural sheetlet angle variation of the *NT* myofiber architecture is significant. In both the apical and equatorial regions, the sheetlet angle declines from approximately $20^\circ$ at the endocardial surface to $-10^\circ$ at the epicardial surface, exhibiting significant negative angles within the midwall area. In basal regions, the sheetlet angle increases from the endocardial surface at $15^\circ$ to nearly $90^\circ$ in the middle of the wall, after which it drops to about $-10^\circ$ on the epicardial surface.

### Global performance metrics

**Fig. 6:**
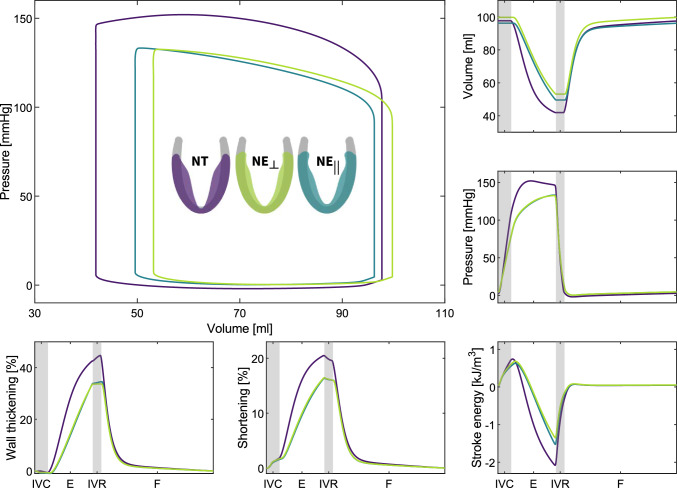
**Temporal evolution ventricular performance for each of the varying myofiber architecture models**. The plots show the pressure–volume relations and the evolution of wall thickening, longitudinal shortening, and the myocardial stroke energy density of the left ventricle with different myofiber architecture during a cardiac cycle. The purple curves show the values for the left ventricle with *NT* myofiber architecture. For $NE_{\perp }$ and $NE_{||}$ myoarchitectures, the values are plotted in green and blue curves, respectively. IVC: isovolumetric contraction, E: ejection, IVR: isovolumetric relaxation and F: filling stage during diastole

**Table 1:** **Global ventricular performance metrics for each of the varying myofiber architecture models.** The columns show the results of global and functional metrics of the left ventricle with different myofiber architectures. Each model was subjected to the same active and passive material parameters and loading conditions

Model	End-diastolic volume (ml)	End-systolic volume (ml)	End-systolic pressure (mmHg)	Stroke volume (ml)	Ejection fraction ($\%$)	Wall thickening ($\%$) ^*^	Shortening ($\%$)^*^	Minimum myocardial stroke energy density ($\mathrm {\frac{kJ}{m^3}}$)^*^
*NT*	97.71	41.91	146	55.80	57	42	20	$-$ 2.08
$NE_{\perp }$	99.76	53.15	132	46.61	46	33	16	$-$ 1.34
$NE_{||}$	96.23	49.58	132	46.65	48	34	16	$-$ 1.52

^*^ Values at end-systole

Figure 6 and Table 1 summarize the impact that *NT*, $NE_{\perp }$, and $NE_{||}$ myofiber architecture differences have on the ventricular pressure–volume relationship, wall thickening, longitudinal shortening, and myocardial stroke energy density, respectively. The ventricular model with the *NT* myofiber architecture has an end-diastolic volume of 97.71 ml and an end-systolic volume of 41.91 ml and the highest stroke volume and ejection fraction of 55.80 ml and $57\%$, respectively. The passive filling and active contraction of a ventricle equipped with a $NE_{||}$ myofiber architecture lead to the smallest end-diastolic ventricular volume of 96.23 ml. In the absence of differences in constitutive tissue properties, hemodynamic loading, or mechanical boundary conditions, the *NT* myofiber model reaches an end-systolic pressure of 146 mmHg, which is approximately $11\%$ higher than the values observed in the $NE_{||}$ and $NE_{\perp }$ myoarchitecture models. We observe peak myocardial work densities at the end of isovolumetric contraction, amounting to 742, 683 and 627 $\mathrm {J/m^3}$ for a ventricle with an underlying *NT*, $NE_{\perp }$, and $NE_{||}$ myofiber architecture, respectively. At end-systole, we notice the myocardial stroke energy density drops to $-2080$ $\mathrm {J/m^3}$ in the *NT* model, while a minimum work density of $-1521$ $\mathrm {J/m^3}$ and $-1349$ $\mathrm {J/m^3}$ is recorded for $NE_{||}$ and $NE_{\perp }$ ventricles. The *NT* model demonstrates the highest ventricular longitudinal shortening and wall thickening during the ejection phase, with maximum values of 20 and 42% at end-systole, respectively. In both $NE_{\perp }$ and $NE_{||}$ models, the ventricular shortening and wall thickening amounted to 16 and 33%, respectively.

### Ventricular wall rotation

**Fig. 7:**
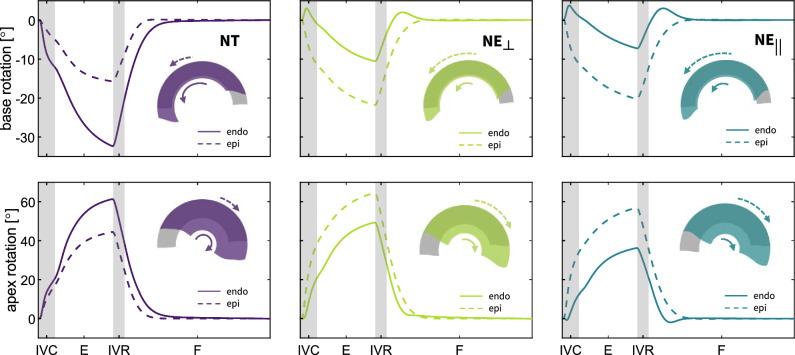
**Temporal evolution ventricular wall rotation for each of the varying myofiber architecture models**. The rotation of the endocardium and epicardium at base and apex is shown by solid and dashed curves, respectively. The upper panels highlight the wall rotation in the basal region, and the lower panels disclose the wall rotation in the apical region. The left ventricle with *NT* myofiber architecture demonstrates a greater rotation of the endocardial wall at both the base and the apex, while an inverse relationship is recorded for the $NE_{\perp }$ and $NE_{||}$ myofiber architectures. IVC: isovolumetric contraction, E: ejection, IVR: isovolumetric relaxation and F: filling stage during diastole

Varying myofiber architectures affect the regional rotation of the ventricular wall. Figure 7 illustrates the rotational dynamics of the endocardial and epicardial wall in both basal and apical regions for each specific myofiber architecture model. Here, positive angles denote a clockwise rotation of the ventricular wall when observed from the base toward the apex. The *NT* ventricular rotation is larger in the endocardial than in the epicardial wall. In contrast, in both $NE_{\perp }$ and $NE_{||}$ myofiber architectures, the epicardial surface exhibits greater rotation compared to the endocardial surface. At peak contraction, we measure an endocardial rotation of $-29^\circ$ and $63^\circ$ in the base and apex for the *NT* architecture, respectively. Concomitantly, the epicardial wall rotates $-15^\circ$ and $44^\circ$ at the base and apex, respectively. In the $NE_{\perp }$ and $NE_{||}$ architectures, the basal rotation of the endocardial wall amounts to $-10^\circ$ and $-7^\circ$, respectively, while the epicardial wall rotates $-21^\circ$ and $-20^\circ$, respectively. In the apex, we observe ventricular wall rotations up $64^\circ$ and $56^\circ$, respectively, for the epicardial wall and $50^\circ$ and $35^\circ$, respectively, for the endocardial wall.

### Sheetlet mobility

**Fig. 8:**
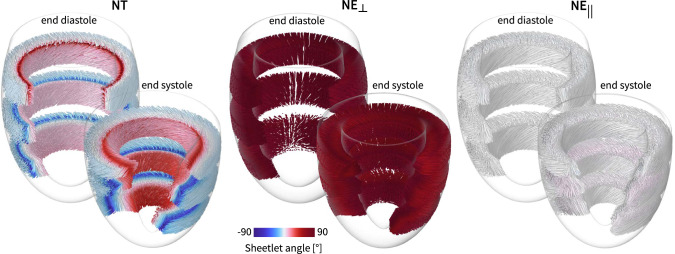
**Sheetlet mobility for each of the varying myofiber architecture models**. The streamlines illustrate the sheetlet orientation at end-diastole and end-systole in three apicobasal sections for the *NT*, $NE_{\perp }$, and $NE_{||}$ myoarchitecture models, respectively. The sheetlets in the left ventricle with *NT* myofiber architecture rotate in a longitudinal-to-circumferential fashion from end-diastole to end-systole. The left ventricle with $NE_{\perp }$ myoarchitecture exhibits a circumferential-to-longitudinal sheetlet rotation during contraction. Sheetlet mobility remains limited in the $NE_{||}$ myoarchitecture models

Figure 8 depicts the sheetlet orientation at end-diastole and end-systole in three different apicobasal regions across the varying myofiber architecture models. The *NT* myofiber ventricle undergoes notable transmural sheetlet tilting (low-to-high sheetlet angle value evolution, see Fig. A2) from end-diastole to end-systole. More specifically, the sheetlet angles near endocardial wall increase from end-diastolic values of $13^\circ$, $10^\circ$, and $14^\circ$ to end-systolic values of $29^\circ$, $39^\circ$, and $52^\circ$ at the basal, equatorial, and apical regions, respectively. These longitudinal-to-circumferential rotations are the highest in the midwall and endocardial layers and remain low in the epicardial region. In the $NE_{\perp }$ myoarchitecture model, we observe complete opposite sheetlet mobility trends. The $NE_{\perp }$ myofiber ventricle undergoes notable transmural sheetlet detilting (high-to-low sheetlet angle value evolution, see Fig. A2.) from end-diastole to end-systole. In this myoarchitecture model, the sheetlet angles decrease from $\pm 90^\circ$ to $\pm 60^\circ$ in the equatorial and apical sections of the epicardial wall. In the endocardial wall, sheetlet mobility is negligible. Lastly, in the $NE_{||}$ myocarchitecture model, the sheetlet angles remain $\pm 0^\circ$ thoughout the whole cardiac cycle in most regions. The maximum observed sheetlet mobility in this model only amounted to $5^\circ$.

### Local tissue mechanics

**Fig. 9:**
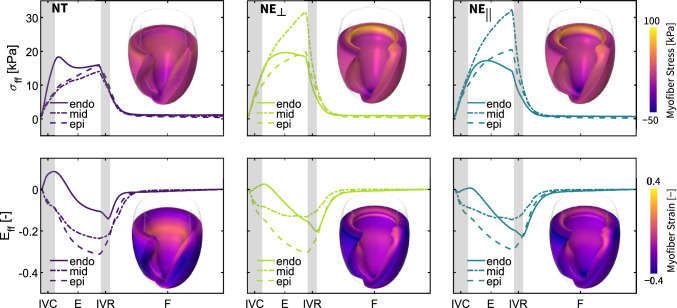
**Spatiotemporal myofiber Cauchy stress and Green–Lagrangian myofiber strain evolution for each of the varying myofiber architecture models**. The top panels showcase the temporal evolution of volume-averaged myofiber stress $\sigma _{ff}$ and the bottom panels illustrate the temporal evolution of the volume-averaged Green–Lagrangian myofiber strain $E_{ff}$. These average stress and strain evolution are shown for three subregions: the endocardial zone in a solid line, the midwall region in a dash–dot line, and the epicardial zone with a dashed line, respectively. Each panel also depicts the spatial distribution of myofiber stress (top panels) and Green–Lagrangian myofiber strain (bottom panels) at end-systole. IVC: isovolumetric contraction, E: ejection, IVR: isovolumetric relaxation and F: filling stage during diastole

Figure 9 outlines the impact that varying myofiber architectures have on the local mechanical behavior of cardiac tissue. More specifically, the three upper panels show the temporal evolution of the myofiber stress in the endocardium, midwall, and epicardium for *NT*, $NE_{\perp }$, and $NE_{||}$ myoarchitectures, respectively. In the *NT* endocardium, we compute myofiber stresses up to 17.25 kPa during isovolumetric contraction and as low as 1.12 kPa at end-diastole. In the *NT* midwall and epicardium, the myofiber stress rises to 14.00 kPa and 15.86 kPa, respectively. Compared to the *NT* myofiber architecture, our results showcase substantially increased peak myofiber stresses in both the $NE_{\perp }$ and $NE_{||}$ myofiber architecture models. In the midwall, we record myofiber stresses up to 31.31 kPa and 32.44 kPa for the $NE_{\perp }$ and $NE_{||}$ models, respectively. The spatial distribution of myofiber stresses at end-systole, shown in the insets of the top panel in Figure 9, further highlights the pronounced stress differences between ventricles with nested toroidal and nested ellipsoidal myofiber architectures. The bottom row plots of Fig. 9 depict the temporal Green–Lagrangian myofiber strain evolution during a cardiac cycle for each of the myoarchitectures studied. Myofiber strains are computed relative to the end-diastolic state. During the isovolumetric contraction stage, endocardial myofibers are tensioned while the midwall and epicardial myofibers are contracting. At the end of this stage, we measure endocardial, midwall, and epicardial myofiber strains of 0.08, $-0.10$, and $-0.10$ for the *NT* myoarchitecture model, 0.03, $-0.08$, and $-0.14$ for the $NE_{\perp }$ myoarchitecture model, and 0.01, $-0.08$, and $-0.13$ for the $NE_{||}$ myoarchitecture model, respectively. In addition, at maximum contraction we measure endocardial, midwall, and epicardial myofiber strains of $-0.10$, $-0.23$, and $-0.30$ for the *NT* myoarchitecture model, $-0.16$, $-0.12$, and $-0.30$ for the $NE_{\perp }$ myoarchitecture model, and $-0.20$, $-0.13$, and $-0.29$ for the $NE_{||}$ myoarchitecture model, respectively.

## Discussion

In this study, we introduced a novel biomechanical model of the left ventricle that incorporates a myofiber architecture inspired by Streeter’s nested tori conjecture. We developed a method to define nested toroidal myofiber and sheetlet distributions that are continuous from the inner to the outer wall, by representing the left ventricular geometry as a series of nested toroidal surfaces. On each toroidal surface, myofibers follow torus knot trajectories, while sheetlets form smoothly twisted surfaces that span from the outermost to the innermost layer. Unlike existing nested ellipsoidal rule-based methods, our approach does not require the explicit prescription of transmural rules for fiber and sheetlet angles. Instead, these orientations emerge naturally from the structural geometry of the ventricle. Recognizing the intrinsic coupling between fibers and sheetlets, we interpret sheetlets as twisted layers that reflect gradual spatial changes in fiber orientation across the myocardial wall (Gilbert et al. 2007). We compared the deformation behavior and functional performance of this nested tori model against conventional myofiber architecture models and identified key structural and mechanical differences introduced by this geometry-driven representation relative to the widely used nested ellipsoidal approaches.

**Nested tori conjecture of the heart muscle.**

The architecture of cardiac muscle has long captivated researchers and is often described as the *Gordian knot* in anatomy. In their seminal work, Streeter et al. (1969) quantitatively described the double helix organization of cardiomyocytes in the canine left ventricle. They showed that fiber angles change gradually across the wall thickness, ranging from left-handed orientations of approximately $+60^{\circ }$ in the subendocardium to right-handed angles of about $-60^{\circ }$ in the subepicardium. Although early hypotheses assumed that fibers aligned parallel to the heart’s inner and outer walls, later studies by Streeter and colleagues revealed that myofibers follow geodesic trajectories across a family of nested toroidal surfaces within the myocardial wall (Daniel 1979). This insight aligns with historical anatomical models, including Krehl’s *Triebwerk* concept (Krehl 1891) and Thomas’s *powerhouse of the heart* (Thomas 1957), which proposed a central cylindrical structure that remains after removing specific myocardial regions. In this model, outer layer fibers spiral counterclockwise from base to apex, then penetrate inward near the apex, and continue spiraling back toward the base. The proximity of these penetration points to the apex correlates with how superficially the fibers are located on the outer wall (Peskin 1989). Streeter’s team further showed that the thickest part of the myocardial wall coincides with the center of the toroidal structure, where the innermost torus reduces to a single circumferential fiber loop. Recent anatomical and imaging studies have supported this nested toroidal organization, demonstrating smooth continuity of myofibers across transmural layers using both dissection and advanced imaging techniques (Jouk et al. 2007; Jouk and Usson 2021; Sanchez-Quintana et al. 1995; Grant 1965; Smerup et al. 2009).

**Our nested tori surrogate model produces realistic myofiber architectures.**

Figure 5 demonstrates that the transmural variation in the myofiber helix angle in our nested torus *NT* model closely resembles that of the nested ellipsoidal $NE_{\perp }$ and $NE_{||}$ models. The nonlinear S-shaped profile of the helix angle across the wall in the *NT* model is consistent with reports for healthy human myofiber architecture (Lombaert et al. 2012; Rohmer et al. 2007). While we selected specific rotational cadence function parameters and morphing settings based on initial physiological and geometric considerations, our NT framework remains inherently flexible. By varying these rotational cadence parameters and our morphing rules, we can model a wide range of fiber architectures, including alternative helix angle distributions reported in the literature. A systematic exploration of this parameter space in future work may further refine the nested tori architecture and improve alignment with subject-specific or region-specific characteristics of the myofiber helix angle.

Most distinct differences between the *NT* model and both *NE* models emerge in the distribution of the myofiber transverse angle. Quantitative validation of transverse angle patterns remains challenging due to limited and noisy experimental data (Kroon et al. 2009; Pluijmert et al. 2017), and even advanced diffusion tensor imaging techniques have difficulty resolving these small angular variations in both in vitro and in vivo settings (Scollan et al. 1998; Tseng et al. 1999). Interestingly, the transverse angle pattern produced by our nested toroidal approach closely resembles those found in computational studies that performed local adaptations of myofiber orientation to minimize fiber–cross-fiber shear (Kroon et al. 2009; Pluijmert et al. 2017), as well as studies that optimized the transverse myofiber angle distribution to minimize regional differences in fiber strain at the onset of ejection (Rijeken et al. 1997; Kerckhoffs et al. 2003). Notably, these studies reported transverse angles that approach zero at both the endocardium and epicardium, with a peak of about $5^{\circ }$ near the base and about $15^{\circ }$ near the apex, similar to the distribution observed in our *NT* model.

With regard to sheetlet angles, many computational studies have adopted a wall-perpendicular orientation, motivated by histological analyses, as implemented in our $NE_{\perp }$ model (LeGrice et al. 1995). Alternatively, some researchers favor a wall-parallel configuration, supported by diffusion tensor imaging data that suggest low sheetlet angles at end-diastole (Hales et al. 2012; Nielles-Vallespin et al. 2017). Prior studies have shown that sheetlet angles can reverse sign from endocardium to epicardium, with abrupt transitions at different transmural locations depending on apicobasal position (Rohmer et al. 2007). Our nested tori model captures similar behavior, with sharp shifts in sheetlet angle arising at differing transmural and apicobasal positions. We attribute these transitions to the accordion- or zigzag-like configuration evident in projections of sheetlet vectors on radial longitudinal slices (see Fig. 5). This behavior is consistent with observations from histological and diffusion tensor imaging studies of myocardial laminar structure (LeGrice et al. 1995; Hales et al. 2012; Rohmer et al. 2007; Costa et al. 1999; Harrington et al. 2005). Key differences lie in the use of the sheetlet vector to define the sheetlet angle in our *NT* model, rather than the sheetlet normal used in diffusion tensor imaging data. In our *NT* model, sharp transitions in the sheetlet angle occur around $\pm 90^\circ$ ranging $[-\pi /2,\pi /2]$, while Rohmer et al. (2007) reported sudden changes from approximately $135^\circ$ to $45^\circ$ centered at $90^\circ$, reflecting their use of the sheet-normal vector to define the sheetlet angle ranging $[0,\pi ]$.

A key structural limitation of the *NE* models is the geometric singularity that appears at the apex, where myofibers converge to a single point on nested ellipsoidal surfaces (Fig. 2). This singularity can lead to stress accumulation and unrealistic deformation patterns in computational simulations. In contrast, the *NT* model avoids this issue entirely, as its fiber field in continuous by construction on nested toroidal surfaces, smoothly transitioning across transmural and apicobasal directions without singularities.

Altogether, these findings suggest that our nested toroidal myoarchitecture offers a structurally and functionally realistic representation of myocardial organization. It captures key trends in helix, transverse, and sheetlet angles reported in the literature, while resolving important geometric limitations present in traditional rule-based ellipsoidal models. As a result, it provides a promising foundation for physiologically informed computational simulations of cardiac mechanics.

For simplicity, the present study employed an idealized left ventricular geometry. A natural next step is to adopt the pretzel geometry instead of a simple torus (Jouk et al. 2007) to extend our nested myofiber architecture modeling to idealized biventricular geometries. To move beyond idealized geometries and generate subject-specific myofiber fields, we envision the use of large deformation diffeomorphic metric mapping (Moscoloni et al. 2025a, b). This framework provides a smooth and invertible transformation between a template geometry and a patient-specific biventricular mesh. Such a transformation enables the morphing of myofiber directions from the template to the patient geometry while preserving topological and anatomical coherence. This approach would offer a principled and automated route for constructing realistic, patient-specific fiber architectures that retain the nested tori organization proposed in this study. These extensions would also support validation against high-resolution diffusion tensor magnetic resonance imaging (DT-MRI) datasets, where such data are available. However, we note that DT-MRI remains limited in its ability to resolve sheetlet orientations and transverse myofiber angles, particularly in compact regions like the apical myocardium. This difficulty stems from the closeness of the secondary and tertiary eigenvalues, rendering the differentiation of these orientations from one another challenging (Gilbert et al. 2007). Invasive dissection is not a viable alternative either, as physical slicing disrupts the tissue’s three-dimensional laminar structure. Our *NT* nested tori model also creates exciting opportunities for future comparison against *NE* nested ellipsoidal architectures that explicitly incorporate transmural variations in sheetlet orientation (Carapella et al. 2014; Gültekin et al. 2016; Eriksson et al. 2013; Nikou et al. 2016). We note that a sufficiently complex Laplace–-Dirichlet rule-based method could, in principle, approximate similar fiber and sheetlet patterns to our *NT* model in the basal region. Extending such comparisons to anatomically realistic geometries could yield new functional insights and further highlight the advantages of the *NT* architecture. Once realistic geometries are in place, a promising avenue for future research will be to investigate the differences between healthy and diseased myofiber structures, following approaches similar to those in Grosberg and Gharib (2009).

**Our nested tori surrogate model captures physiological sheetlet mobility throughout the cardiac cycle.**

The dynamic mechanism of myocardial sheetlet sliding transforms ventricular wall shortening and twist into large shear deformations, which are crucial for wall thickening during systole and fundamental to cardiac pumping function (Nielles-Vallespin et al. 2017; Wilson et al. 2022; Zheng et al. 2023; Costa et al. 1999). As shown in Fig. 8, the $NE_{||}$ model, which assumes a zero sheetlet angle at end-diastole, exhibits minimal sheetlet mobility. This finding is consistent with recent computational studies that emphasize the importance of nonzero diastolic sheetlet angles in enabling effective sheetlet reorientation and sliding during contraction (Zheng et al. 2023).

In contrast, the wall-perpendicular sheetlets in the $NE_{\perp }$ model display an end-diastolic to end-systolic transmural sheetlet detilting near the epicardial wall, where sheetlet angles decrease from $+90^\circ$ in diastole to approximately $+60^\circ$ in systole. However, several studies have shown that transmural sheetlet tilting, defined as an increase in sheetlet angle from low values in diastole to high values in systole, provides the microstructural dynamic deformation basis of the myocardium (Nielles-Vallespin et al. 2017; Harrington et al. 2005; Costa et al. 1999). This pattern, often described as a zigzag linkage or accordion-like configuration, enables a level of wall thickening that far exceeds the contractile capacity of individual cardiomyocytes and contributes significantly to overall cardiac performance.

Our nested tori *NT* myofiber architecture embodies this zigzag configuration (see black arrows in Fig. 5 and streamlines in Fig. 8), exhibiting a transmurally tilting sheetlet mobility, particularly in the midwall and near the endocardial wall. In our simulations, sheetlet angles in the *NT* model predominantly change from low absolute values in diastole (white-colored streamlines in Fig. 8) to high absolute values in systole (red and blue-colored streamlines), with a maximum tilting of up to $38^\circ$. This compares reasonably well with the average sheetlet mobility $+46^\circ$ reported in vivo by Nielles-Vallespin et al. (2017), who measured sheetlet angles ranging from $+11^\circ$ to $+15^\circ$ in diastole and $+52^\circ$ to $+63^\circ$ in systole. While their values reflect global myocardial averages, our result captures a local maximum, primarily in the midwall and near the endocardial wall. The observed localized tilting points to region-dependent dynamics that could be further elucidated by exploring the parameters of the *NT* myoarchitecture and adopting a more anatomically realistic ventricular geometry, both of which may influence sheetlet mobility. This alignment with experimental data underscores the ability of the *NT* model to reproduce physiologically relevant microstructural dynamics. Furthermore, the endocardial wall in the *NT* model exhibits greater sheetlet mobility than the epicardial wall, reflecting the large endocardial sheet-normal shear reported in previous studies (Costa et al. 1999).

Together, these findings highlight the contrasts in sheetlet mobility between our nested toroidal *NT* and current nested ellipsoidal *NE* myoarchitecture surrogate models. By aligning our results with diffusion tensor imaging data and incorporating key mechanical behaviors, our *NT* myofiber architecture offers a more realistic and functionally accurate representation of cardiac tissue dynamics.

**A nested tori myoarchitecture leads to uniform stress distributions and efficient ventricular pump function.**

Our findings demonstrate that the *NT* model facilitates larger contraction during ejection compared to the *NE* models, despite the incorporation of the exact same maximum contractility in all three models. We can attribute the increased deformation to the presence of a nonzero transverse myofiber angle and the smooth continuity of myofibers between different transmural and apicobasal layers in our *NT* model, which contribute to more efficient ventricular function.

During diastole, the $NE_{||}$ model behaves the stiffest due to myocardial tissue’s decreased compliance in the sheetlet direction, resulting in a greater longitudinal stiffness and a lower end-diastolic volume. In contrast, the $NE_{\perp }$ model, with wall-perpendicular sheetlet orientations, exhibits the highest end-diastolic volume. Although the *NT* model has a higher proportion of nearly wall-parallel sheetlets, its end-diastolic volume is closer to that of $NE_{\perp }$, explained by higher sheetlet mobility in *NT* that facilitates wall thinning and longitudinal lengthening during pressurization.

During systole, two architecture-related mechanisms lead to greater deformation and increased pump function in the *NT* model. First, the nonzero *NT* myofibers’ transverse angle introduces a radial component to the contractile force, contributing to wall thickening and resulting in increased stroke volume and higher end-systolic pressures (Kroon et al. 2009; Pluijmert et al. 2017; Bovendeerd et al. 2009). Second, greater sheetlet sliding in *NT* decreases myocardial resistance to deformation and myofiber stress, permitting increased contraction. Again, this sheetlet sliding action enhances contractile performance by allowing wall thickening beyond that achievable by individual cardiomyocytes.

Figure 9 illustrates that the overall myofiber stress in the *NT* model at end-systole is lower and distributed more uniformly in transmural and apicobasal directions compared to the $NE_{\perp }$ and $NE_{||}$ models. The *NE* models exhibit higher stress accumulation near the base and apex, possibly due to fiber discontinuities and singularities in these regions, as previously discussed. Our observations align well with other studies (Pluijmert et al. 2017; Kroon et al. 2009) reporting that the presence of a nonzero transverse angle leads to a more homogeneous distribution of myofiber stress and strain. The uniform stress distribution in *NT* can be further correlated with the intrinsic differences in wall rotation patterns and higher sheetlet mobility with respect to the *NE* models.

In vivo measurements indicate that the myofiber strain distribution through the ventricular wall at peak systole is relatively uniform, with a mean value of about $-0.14$ (Moulin et al. 2021). In contrast, all of our models predict a more negative epicardial strain, reaching values as low as $-0.30$. This discrepancy likely arises because the absence of the right ventricle in our models allows the epicardial layer excessive freedom to move and contract. In the *NT* model, myofiber strain in the midwall lies between that of the endocardial and epicardial layers. Because the endocardial and epicardial fibers form a continuous network, greater contraction of the epicardial fibers can exert tension on the endocardial fibers, thereby improving their ability to shorten. In vivo studies on transmural dispersion of myofiber strain (Ashikaga et al. 2007) showed that epicardial activation lags behind endocardial activation, stretching epicardial fibers during isovolumetric contraction and triggering earlier endocardial relaxation during the subsequent isovolumetric relaxation. This delay narrows the strain difference between the two layers by end-systole, while mid-wall fibers experience the greatest shortening. By contrast, all three of our models exhibit the opposite endocardial–epicardial strain pattern. To reconcile this discrepancy, future work will introduce layer-specific activation delays, explicitly representing transmural heterogeneity in myofiber contraction timing (Peirlinck et al. 2021, 2022). Based on our current results, we anticipate that implementing the experimentally observed delay in our *NT* model will converge endocardial and epicardial strain curves and maintain the dominant contraction of mid-wall fibers. However, in both our *NE* variants the midwall fibers contract less than the endocardial and epicardial layers at end-systole, contradicting the observed transmural fiber strain pattern. Moreover, the endocardial fibers in $NE_{||}$ shorten more than those in $NE_{\perp }$, presumably because the tissue in $NE_{||}$ is less stiff in the radial direction, allowing greater fiber shortening.

Additionally, the *NT* model properly captures in vivo measured relative rotations of the endocardial and epicardial wall (Chitiboi et al. 2016; MacGowan et al. 1996; Rademakers et al. 1992). Left ventricular torsion features distinct apical counterclockwise and basal clockwise rotations, which are both more pronounced in the endocardium than in the epicardium. This transmural gradient induces circumferential–radial shearing, facilitating wall thickening by allowing myocardial sheetlets to slide over one another. Although subendocardial fibers can oppose torsion near the apex and base, myofibers’ transverse orientation may transmit epicardial forces more effectively to the endocardium, explaining the observed rotational gradients. Further investigation into this fiber torsion interplay is crucial for understanding normal left ventricle function and its alterations in disease (Young and Cowan 2012). In the *NE* models, the singularity at the apex leads to higher rotation of the epicardial wall compared to the endocardial wall. The *NT* architecture, with its continuous fiber pathways and transverse angles, allows for the formation of vortex patterns at different apicobasal levels, facilitating effective transmission of forces from the epicardium to the endocardium and back. Near the apex and base, the presence of nonzero transverse angles significantly affects the relative rotation between the endocardium and epicardium, contributing to physiological transverse shear within the myocardium (Bovendeerd et al. 2009; Ubbink et al. 2006). While the *NT* model shows improved rotation patterns, all three models demonstrate excessive apical rotation exceeding physiological values, likely due to the simplified left ventricular geometry used and the absence of the right ventricle, which affects rotational dynamics (Eriksson et al. 2013).

To better understand the effect of the nested tori myofiber architecture on cardiac performance, we further analyzed the evolution of the average moyocardial work density in the left ventricle. Under the same level of active stress, the *NT* model stores higher myocardial stroke energy density during the isovolumetric contraction phase and exhibits the lowest value at peak contraction among the models. The greater difference in myocardial stroke energy density between pre-ejection and post-ejection phases indicates that more energy is converted into cardiac pump work.

## Conclusion

We developed a novel strategy for modeling the left ventricle’s myofiber architecture using nested toroidal surfaces, providing the first detailed examination of its effects on both local myocardial tissue behavior and global ventricular performance. Structurally, our nested tori *NT* myoarchitecture closely aligns with experimental observations and ensures smooth continuity of myofibers throughout the myocardial wall, overcoming limitations of other state-of-the-art rule-based myofiber architecture surrogate approaches. Functionally, a nested tori myofiber architecture enhances the uniformity of stress distribution and improves the efficiency of ventricular pump function. This study opens new avenues for research in cardiac (patho)physiology and biomimetic engineering, fostering the development of innovative therapeutic strategies and bioinspired technologies that could eventually benefit cardiovascular health.

## Data Availability

No datasets were generated or analyzed during the current study.

## Appendix

**Table A1:** **Geometrical, boundary condition, constitutive, and hemodynamic loading condition parameters**. Summary of all the key parameters employed in our myocardial mechanics simulations, including geometrical dimensions, boundary conditions, both passive and active constitutive material properties, and Windkessel model parameters

Geometry				Passive myocardial tissue			
Parameter	Value	Unit	Description	Parameter	Value	Unit	Description
$r_{endo}$	18	mm	Endo-minor radii	*a*	94.3	$\textrm{Pa}$	Isotropic matrix
$R_{endo}$	50	mm	Endo-major radii	*b*	5.874	–	
$r_{epi}$	33	mm	Epi-minor radii	$a_{f}$	311	$\textrm{Pa}$	Fiber stiffness
$R_{epi}$	60	mm	Epi-major radii	$b_{f}$	11.271	–	
$\eta _{endo}$	[$\frac{\pi }{2}$, $\pi$]	Rad	Endo-angular parameter	$a_{s}$	43.1	$\textrm{Pa}$	Sheetlet stiffness
$\eta _{epi}$	[$\frac{\pi }{10}$ ,$\pi$]	Rad	Epi-angular parameter	$b_{s}$	9.772	–	
$P_{0}$	[32.51, 10.24]	mm	Bézier point (r,z)	$a_{fs}$	25.4	$\textrm{Pa}$	Fiber sheet shear stiffness
$P_{1}$	[30.93, 21.12]	mm	Bézier point (r,z)	$b_{fs}$	2.405	–	
$P_{2}$	[29.10, 22.68]	mm	Bézier point (r,z)	$\kappa$	$10^6$	$\textrm{Pa}$	Volumetric penalty
$P_{3}$	[26.95, 22.83]	mm	Bézier point (r,z)	$\eta$	10	$\mathrm {Pa.s}$	Viscosity
$P_{4}$	[24.56, 23.01]	mm	Bézier point (r,z)	Active myocardial tissue			
$P_{5}$	[21.77, 21.45]	mm	Bézier point (r,z)	$\alpha _{max}$	5	–	Activation rate
$$P_{6}$$	[19.95, 18.15]	mm	Bézier point (r,z)	$$\alpha _{min}$$	$$-30 $$	–	Deactivation rate
$P_{7}$	[18, 14.60]	mm	Bézier point (r,z)	$\sigma _{0}$	50	$\textrm{kPa}$	Ventricular contractility
$P_{8}$	[18, 0]	mm	Bézier point (r,z)	$t_{dias}$	1	$\textrm{s}$	Start of diastole
Boundary conditions				$t_{sys}$	0.676	$\textrm{s}$	Start of systole
$r_{peri}$	35	mm	Peri-minor radii	$\gamma$	0.005	$\textrm{s}$	Steepness
$R_{peri}$	60	mm	Peri-major radii	Windkessel model			
$\alpha ^n_{epi}$	$10^8$	$\mathrm {\frac{N}{m^3}}$	Peri-normal stiffness	$R_{a}$	$8\times 10^6$	$\mathrm {\frac{Pa.s}{m^3}}$	Arterial resistance
$\beta ^n_{epi}$	5000	$\mathrm {\frac{N.s}{m^3}}$	Peri-normal damping	$R_{p}$	$3\times 10^8$	$\mathrm {\frac{Pa.s}{m^3}}$	Peripheral resistance
$\alpha ^t_{epi}$	$10^5$	$\mathrm {\frac{N}{m^3}}$	Peri-tangential stiffness	$R_{v}$	$1\times 10^6$	$\mathrm {\frac{Pa.s}{m^3}}$	Venous resistance
$\beta ^t_{epi}$	1000	$\mathrm {\frac{N.s}{m^3}}$	Peri-tangential damping	$C_{a}$	$8\times 10^{-9}$	$\mathrm {\frac{m^3}{Pa}}$	Arterial compliance
$\alpha _{base}$	$10^5$	$\mathrm {\frac{N}{m^3}}$	Base stiffness	$C_{v}$	$5\times 10^{-8}$	$\mathrm {\frac{m^3}{Pa}}$	Venous compliance
$\beta _{base}$	1000	$\mathrm {\frac{N.s}{m^3}}$	Base damping	$p_{lv,i}$	10	mmHg	Initial ventricular pressure
